# The Irvine, Beatties, and Bresnahan (IBB) Forelimb Recovery Scale: An Assessment of Reliability and Validity

**DOI:** 10.3389/fneur.2014.00116

**Published:** 2014-07-07

**Authors:** Karen-Amanda Irvine, Adam R. Ferguson, Kathleen D. Mitchell, Stephanie B. Beattie, Amity Lin, Ellen D. Stuck, J. Russell Huie, Jessica L. Nielson, Jason F. Talbott, Tomoo Inoue, Michael S. Beattie, Jacqueline C. Bresnahan

**Affiliations:** ^1^Brain and Spinal Cord Injury Center, Department of Neurological Surgery, University of California San Francisco, San Francisco, CA, USA

**Keywords:** spinal cord injury, recovery of function, forelimb functional task, reliability, validity

## Abstract

The IBB scale is a recently developed forelimb scale for the assessment of fine control of the forelimb and digits after cervical spinal cord injury [SCI; (1)]. The present paper describes the assessment of inter-rater reliability and face, concurrent and construct validity of this scale following SCI. It demonstrates that the IBB is a reliable and valid scale that is sensitive to severity of SCI and to recovery over time. In addition, the IBB correlates with other outcome measures and is highly predictive of biological measures of tissue pathology. Multivariate analysis using principal component analysis (PCA) demonstrates that the IBB is highly predictive of the syndromic outcome after SCI (2), and is among the best predictors of bio-behavioral function, based on strong construct validity. Altogether, the data suggest that the IBB, especially in concert with other measures, is a reliable and valid tool for assessing neurological deficits in fine motor control of the distal forelimb, and represents a powerful addition to multivariate outcome batteries aimed at documenting recovery of function after cervical SCI in rats.

## Introduction

Motor function loss is a major consequence of spinal cord injury (SCI) and has been the focus of experimental studies for over a century. Most studies have used thoracic injury models and assessed locomotor function as the primary outcome measure. A number of cervical injury models have been developed (3–9), and are being used more frequently due to the understanding that the majority of SCI occurs at this level in the human population (10). Individuals with cervical injuries are reported to be most interested in the reinstatement of hand function (11), and hence outcome measures focused on recovery of forelimb use are becoming more commonplace.

In our attempts to model cervical SCI, we chose to use unilateral injuries to reduce the burden of neurological deficits, including bladder dysfunction and quadriplegia. Prior work (4) had shown the feasibility of this approach. We used the well-established MASCIS injury device for the early studies (6), but are now using the IH device (2, 12) due to its currently widespread use in the SCI research community. We selected outcome measures that evaluated spontaneously expressed behaviors, thus reducing training requirements and food deprivation since weight loss is a consistent consequence of SCI. In our initial studies (6), we measured paw placement during vertical exploration as originally described by Schallert et al. (13) for assessing forebrain injuries, grooming as originally described by Bertelli and Mira (14) for assessing brachial plexus injuries, over-ground locomotion in an open field and on the Catwalk apparatus (Noldus Information Technology, Sterling, VA, USA), and locomotion on a horizontal ladder (4, 15, 16). Performance on most of these measures reflected graded injury effects, and using principle components analysis (PCA), these behavioral outcomes were seen to co-vary with biomechanical and anatomical descriptors of the lesion (2). However, what was missing in this battery of tests was an assessment of distal forelimb and digit function.

Food retrieval and manipulation for consumption is a critical behavior that is spontaneously expressed in all individuals across mammalian species, and requires involvement of both proximal and distal forelimb. A novel task involving food manipulation was described by Allred et al. (17) and was based on the observations of Whishaw and Coles (18). In this task, pasta is presented to rats for eating and forelimb use is assessed during consumption. This test was sensitive to a number of forebrain injuries. In our initial attempts to use this test with spinal cord injured animals, we discovered that our rats were not particularly interested in eating pasta but would readily consume sugared cereal, which is available in a variety of shapes of consistent size. The manipulation of these cereal pieces was observed to involve detailed movements of the forelimbs and digits as the rats rotated the cereal pieces and somewhat systematically bit off small chunks to eat. Therefore, we attempted to evaluate the movements that were used to manipulate these food items while recovering from unilateral cervical contusion injuries. The first attempt to establish a recovery scale was presented in a video and manuscript (1) describing the methods, and termed the “IBB.” The scale was generated by characterizing the movements made during cereal eating over the post-SCI recovery period, and assigning an ascending series of numbers for each functional set, and adjusting the scale until it reflected a sequential representation of the recovery (1). This procedure was based on our prior experience in developing and testing the Basso–Beattie–Bresnahan (BBB) locomotor rating scale (19). In that effort, we used an iterative process to construct an ordinal scale that withstood the test of inter-rater reliability (IRR) and construct validity (20, 21). The usefulness and metric properties of motor outcome scales are not always tested or considered in the SCI literature. But in response to suggestions made as more and more laboratories adopted the BBB and more data became available, this scale was modified in light of a growing body of data that suggested the metric properties were not optimized (22). A similar approach has been taken in the construction of scales for walking in human SCI patients (23). Similarly, in the present paper, we describe modifications to the original IBB scale based on our iterative evaluation of its usefulness and attempt to establish its validity and reliability. In addition, using the syndromics approach described recently for cervical SCI (2), we are now able to evaluate the relationship of this new outcome scale to other forelimb functional tests currently in use in our laboratory and in the field.

We first provide a brief history of the scale and metric properties analysis that guided its initial development. We then present results of IRR testing across a group of 9–10 novice and expert raters, and propose some minor revisions that improve reliability. Finally, we address the issue of validity (face, concurrent, predictive, external, and construct validity) for the IBB scale.

The results demonstrate that the IBB is a reliable and valid scale that is sensitive to injury severity and recovery over time. In addition, the IBB correlates with other outcome measures and is highly predictive of biological measures of tissue pathology. Multivariate analysis using PCA demonstrates that the IBB is highly predictive of the syndromic outcome after SCI, and is among the best predictors of bio-behavioral function, that is, there is good evidence of construct validity. Altogether, the data suggest that the IBB, especially in concert with other measures, is a reliable and valid tool for assessing neurological deficits in fine motor control of the distal forelimb, and represents a powerful addition to multivariate outcome batteries aimed at documenting recovery of function after cervical SCI in rats. Further, the similarities of “hand function” across rodents and primates may make such measures as this especially important in translating therapeutic strategies from rodent studies to clinical studies in man.

## Materials and Methods

### Animals

Long Evans and Sprague Dawley rats aged 77–87 days at the time of injury were used in the initial scale development and validity testing (*N* = 70). All experiments adhered to the National Institutes of Health Guide for the Care and Use of Animals and were approved by the Institutional Animal Care and Use Committee (IACUC) at the University of California San Francisco (UCSF). For many of the subjects, the primary data on non-IBB outcomes have been presented elsewhere as part of recently published papers (2, 24). These data are re-plotted here (with permission) for the purposes of comparative (concurrent) validity testing of the IBB.

### Surgical procedures for cervical SCI

All surgical procedures were performed aseptically as described previously (6). Briefly, animals were anesthetized with Ketamine HCL (80 mg/kg, Abbott Laboratories, North Chicago, IL, USA) and Xylazine (20 mg/kg, TraquidVed, Vedco Inc., St Joseph, MO, USA) intraperitoneally (ip) or with isoflurane before surgery. A dorsal, midline skin incision was made, the skin dissected, and the trapezius muscle was cut just lateral to the midline from C2 to T2. Spinous processes from C4 to T1 were exposed and a C5 dorsal laminectomy was performed to expose the entire right side and most of the left side of the underlying spinal cord. Contusion injuries were produced using the Infinite Horizon Impactor (Precision Systems and Instrumentation LLC, Fairfax, VA, USA) with a modified impactor tip 2 mm in diameter, with a force of 75 (mild) or 100 (moderate) kdynes. Cord hemisections were performed in a separate group of animals at the same vertebral level by inserting the tip of a #11 blade at the midline and sweeping laterally to cut all fibers of the hemi-cord. The sham group of animals underwent the laminectomy without SCI. The wound was closed in anatomical layers. The analgesic, buprenorphine (0.05 mg/kg, Buprenex, Hospira, IL, USA), and the antibiotic, Cefazolin (50 mg/kg, Henry Schein, Melville, NY, USA) were administered, and the animal recovered overnight in an incubator (Thermocare^®^, Intensive Care Unit with Dome Cover; Thermocare, Incline Village, NV, USA). All animals were inspected daily for wound healing, weight loss, dehydration, autophagia, and discomfort. Appropriate veterinary care was provided when needed.

### Surgical procedures for traumatic brain injury

A controlled cortical contusion injury (CCI) was produced using a device that has been described in detail elsewhere (25). Briefly, rats were mounted in a Kopf stereotaxic frame under isoflurane anesthesia. A unilateral craniectomy (6.0 mm diameter) between 3.0 mm posterior and 3.0 mm anterior to bregma, and between 1.0 and 7.0 mm lateral to bregma was produced using a high-speed drill. CCI was produced using a 5.0 mm diameter impactor with a convex tip (Custom Design & Fabrication, Inc., Sandston, VA, USA), oriented perpendicular to the cortical surface. The cortex was compressed to a depth of 2.0 mm at 4.0 m/s velocity with a dwell time of 150 ms. Sham animals received the craniectomy only. During the surgical procedure, heart rate and blood oxygenation were monitored with a Mouse Ox™ pulse-oximeter (Torrington, CT, USA); temperature was monitored and maintained at 37.5°C. The injury sites were closed and the animals were recovered in an incubator (Thermocare^®^, Intensive Care Unit with Dome Cover; Thermocare, Incline Village, NV, USA).

### Combined SCI + TBI

In animals with both traumatic brain injury (TBI) and SCI, both surgical sites were prepared and then the TBI was performed followed by the SCI. All other aspects of the procedure were as described above and previously (24).

### Behavioral testing

All behavioral testing for the IRR and validity testing was performed by raters who were blind to the experimental condition. Testing was typically performed pre-operatively and on post-operative days 2, 7, 14, 21, 28, 35, and 42 after injury.

#### Forelimb testing using the Irvine, Beattie, and Bresnahan (IBB) Scale

**Figure 1:**
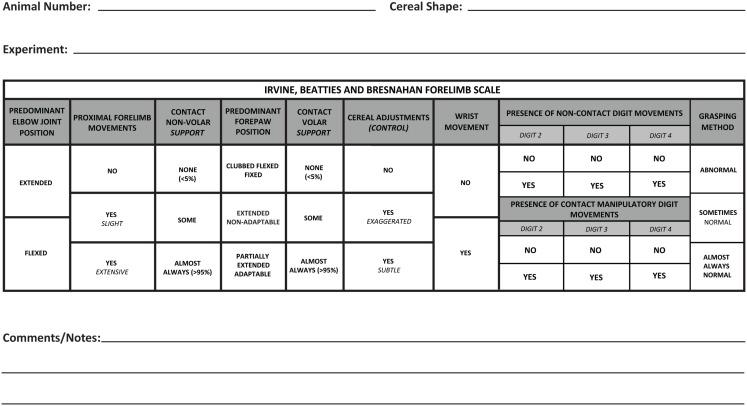
**The revised scoring sheet with individual categories that accompanies the Irvine, Beatties, and Bresnahan (IBB) forelimb scale**. The first half of the sheet represents recovery of proximal forelimb function and the latter part of the sheet focuses on recovery of the forepaw.

Rats were given pieces of cereal in their home cage twice daily beginning as soon as they entered the lab. Forelimb function was assessed while rats were eating cereal as described previously (1). Briefly, rats were individually placed in a Plexiglas cylinder (diameter = 20 cm; height = 46 cm) or in their home cage and given spherical- and donut-shaped pieces of cereal (“Reeses Puffs™,” The Hershey Co., and “Froot Loops™,” Kellogg’s Co.) that were of a consistent size and shape prior to the initiation of eating. Rats were not scored when eating cereal pieces that were broken prior to the initiation of testing. Each trial was recorded to allow slow motion HD playback and evaluation of forelimb use. Videos of animals eating the cereal were evaluated using a standardized scoring sheet (Figure 1) to record observations of forelimb behaviors, including joint position, object support, wrist and digit movement, and grasping method used while consuming both cereal shapes. An IBB score was assigned using the 10-point (0–9) ordinal scale for each shape, and the highest score reflecting the greatest amount of forelimb recovery, was assigned.

#### Grooming test

Forelimb grooming function was assessed using a scoring system described previously (6). Cool tap water was applied to the animal’s head and back with soft gauze, and the animal was placed in a clear plastic cylinder (diameter = 20 cm; height = 46 cm) or in their home cage. Grooming activity was recorded with a video camera from the onset of grooming through at least two stereotypical grooming sequences (~2 min). A score was assigned depending on the highest region touched by the hand as follows: 0, no contact with the head; 1, contact with the mouth only; 2 contact with the snout below the eyes; 3, contact with the face from the eye level to below the ears; 4, contact with the ears; 5, contact with the head behind the ears. Slow motion video playback was used to score each forelimb independently by the maximal contact made while initiating any part of the grooming sequence. The animals were tested on day 2 post-operatively, and then at least weekly until sacrifice.

#### Forelimb use during vertical exploration: forelimb asymmetry or cylinder test

Animals were placed in a clear plastic cylinder and spontaneous exploratory behavior was recorded for 5 min. Slow motion video playback was used to determine the number of times the animal placed its left, right, or both hands against the side of the cylinder during weight-supported movements according to previously published criteria (26). Individual placements were scored as either “left” or “right” when 0.5 s or more passed without the other limb contacting the side of the cylinder. If both hands were used for weight-supported movements within 0.5 s of each other, a score of “both” was given. Results are reported as a percentage of contralateral limb use versus total placements and reported as the “paw preference” outcome.

#### Over-ground locomotion

Forelimb use during over-ground locomotion was assessed in an open field. Limb use for stepping was assessed using a simple four-point scale: 0, no use of the forelimb; 1, stepping on the dorsal surface of the paw; 2, stepping on both the dorsal and plantar surface of the paw; 3, stepping on the plantar surface only.

#### CatWalk

The walkway and CatWalk analysis program was used to measure forelimb function during gait as described previously (27). Briefly, animals were trained to cross a glass walkway (120 cm long) with black Plexiglass walls and ceiling. Light transmitted through the walkway floor revealed foot contacts which were captured and collected by a digital video camera placed underneath the runway (for details, see Figure 9). A digital file for each run across the middle 90 cm of the walkway was analyzed using the CatWalk program (version 7). Measurements for locomotion included stride length, print area during maximal contact, and the distribution of total steps among the four limbs. During training, animals were gently guided to make complete passes across the walkway and were reinforced with sugared cereal or access to the home cage. Data were gathered pre-operatively (baseline), and then at 2–3 week-intervals post-operatively. Data were averaged across five runs in which the animal maintained a constant speed across the middle 90 cm of the CatWalk runway.

#### Inter-rater reliability testing protocol

**Figure 2:**
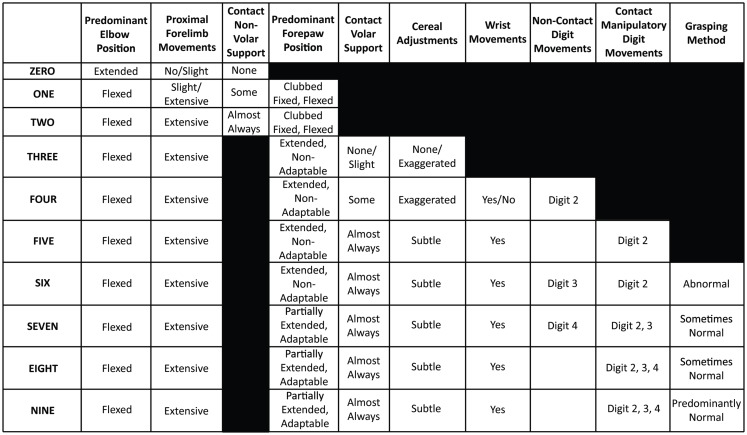
**The score determination guide**. This guide can be used to aid in the selection of the correct IBB score after viewing the video and filling out the IBB score sheet.

Inter-rater reliability was assessed by measuring means and standard deviations of ratings of the same 10 rat videos chosen to represent all parts of the IBB scale, across multiple raters similar to that described for the BBB (21). In the first IRR, nine participants were given an initial IBB training session in which videos of the pattern of recovery in rats with cervical unilateral SCI were shown and the method of scoring using the IBB was explained. The rating of individual rats was then practiced with concurrent discussions, followed by individuals silently rating, and then comparing and discussing scores with those of the trainers. Then each participant was given a CD with ten videos of rats performing at all levels of recovery; each CD presented the videos in a different, randomized order. Also provided to each rater were a set of data recording sheets (Figure 1), a copy of the originally published IBB manuscript and video instructions (1), a set of frequently asked questions with answers, and a score determination guide for ease of assigning scores (Figure 2 shows the revised version). All participants then independently evaluated the 10 videos and assigned IBB scores based on the descriptions provided in Ref. (1). Data sheets were then collected, analyzed, and compared to a consensus score for each rat, arrived at by the original scale developers viewing, discussing, and arriving at a consensus score for each video. This consensus score was determined after all raters (including the experienced raters) had completed and submitted their independent ratings of the videos. The initial IRR test results then were discussed with the participants and problems in recognizing behavioral elements and in assigning scores were identified. Choices, definitions, and the score sheet were then revised to overcome the identified issues for the purpose of improving clarity and consistency in score assignment. Subsequently, a second IRR test was performed approximately 3 months later, with 10 raters most of whom participated in the first IRR test described above, and using the newly revised definitions and the modified score sheet. Consensus scores were determined as in test 1 and individual scores were again assessed for variation from the consensus score as in the first IRR test.

### Histological preparation and morphological analysis

Animals were perfused through the left ventricle of the heart with 4% paraformaldehyde under deep anesthesia with pentobarbital or ketamine–xylazine. The cords were removed and post-fixed in 4% paraformaldehyde for 2 h and then cryoprotected in PBS containing 30% sucrose. A 2 mm block containing the lesion epicenter was then incubated in 100% OCT for 1 h and then mounted in a cryomold (filled with OCT) in coronal orientation and rapidly frozen using dry ice. The blocks were stored at −80°C until sectioning. The cords were cut coronally at 10 μm and every section was retained and mounted. Sections were stained with Luxol fast blue or eriochrome cyanine for myelin/white matter integrity and counterstained with Cresyl violet or neutral red for cell body assessment.

#### Sparing at lesion epicenter

A camera lucida drawing of the section with the largest lesion extent (i.e., the lesion epicenter) was made outlining intact gray and white matter, and the lesion. Pixel counts from digitized drawings in Adobe Photoshop 5.5 (Adobe Systems Inc., San Jose, CA, USA) were used to determine the area of spared tissue for both hemi-cords at the lesion epicenter. The percent sparing for the ipsilateral hemi-cord was determined by dividing the total spared ipsilateral tissue area, spared white matter tissue area, or spared gray matter tissue area, by the same measure from the contralateral hemi-cord [(ipsilateral spared tissue area/contralateral spared tissue area) × 100]. Quantifying pathology in this manner normalized tissue sparing within subjects and corrected for any biological differences in spinal cord size or tissue preparation. Motor neuron counts through the lesion region were performed as in Ferguson et al. (28).

### Statistical analysis

All analyses were performed using SPSS v.19 (IBM) using base, regression, advanced models, and missing values packages. All graphs were generated in Graphpad Prism.

#### Inter-rater reliability assessment

Comparisons across raters were analyzed by assessing individual rater deviations from the “gold standard” or experienced rater-derived consensus scores on the same set of behavioral videos, using the formulas

(1) $$Difference=\sum_{i,j}\left|X_{j}-\mu_{j}\right|$$

and the mean difference score (MDS) is represented by

(2) $$\text{MDS=}\frac{Difference}{n_{i,j}}$$

where *i* = individual rater, *j* = individual rat, *X_ij_* = observed score on rat *j* by rater *i*, μ*_j_* = consensus score on rat *j, n_ij_* = total number of observations by all raters for all rats.

Separate *MDS* values were calculated for expert and novice raters. In addition, MDS values for the novice and expert raters were regressed onto the consensus scores to assess the degree of linear correlation of assessments across raters.

#### Validity assessment

Internal and face validity were examined by testing whether the IBB responded to the impact of graded injury and recovery over time using two-way mixed analysis of variance (ANOVA). In addition, we assessed sensitivity/propriety of applying parametric statistics (e.g., ANOVA) to the IBB by assessing variance-explained (eta squared). Concurrent validity was assessed by correlating the IBB with other more established behavioral measures used by the SCI research community. Predictive validity was assessed by correlating IBB scores with terminal histology. Construct validity was assessed at a multivariate level using exploratory factor analysis using the principal component analysis (PCA) extraction method (2, 29, 30).

## Results

### Initial scaling

Based on general observations of rats with SCI while consuming cereal, we first divided the behaviors into different categories (posture, proximal forelimb joint movement, contact with the food object, digital clubbing, wrist movements, digital movements, and grasping method). These categories were further subdivided into ranks (e.g., no, yes but abnormal, yes but normal) and operational definitions were developed to describe the categories and attributes. Categories were loosely arranged to reflect the sequence of recovery, and scores were assigned (0, 1, 2) to reflect the rank-ordered attributes. Initial scaling involved summation of these ranked features and then the resulting 55-point scale was subjected to evaluation of the metric properties such as score frequency distribution, ordinality, discontinuities, and interval properties (22). This analysis revealed that certain features did not progress in an ordered sequence and further reanalysis revealed problems with reliability and sensitivity that increased measurement error and reduced ordinality. Through this process, we improved the operational definitions of observed behaviors and switched from a summation-based scale to an ordinal scale with fixed definitions of each point. Ultimately, scores were winnowed down to a 10-point (0–9) scale that was published in video format (1). In the present paper, further modifications to the operational definitions are reported to correct for inconsistencies and interpretational difficulties identified during the formal IRR testing analysis as presented below.

### Data record sheet

An initial scoring sheet was developed to use with the IBB for ease of recording observations while viewing subjects eating cereal, and was provided in the original IBB manuscript and video (1). The data sheet was organized from left to right to reflect the course of recovery after SCI, with the earliest behaviors to recover being positioned on the left and the later behaviors on the right. The individual subcategories were organized from top to bottom to reflect less to more recovery. This data sheet was revised to reflect changes resulting from the current analysis as described below; the revised data sheet is now shown in Figure 1.

### Inter-rater reliability

#### Inter-rater reliability test 1

**Figure 3:**
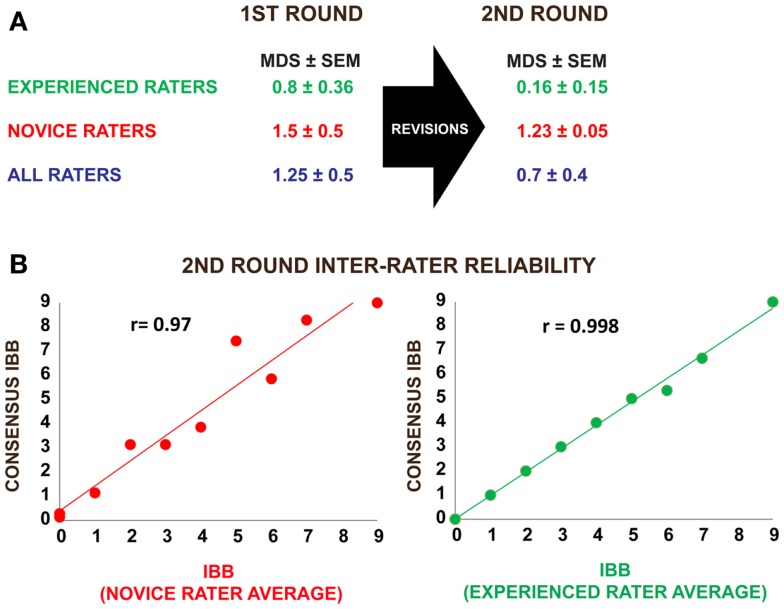
**Results of inter-rater reliability testing using a standardized set of rat behavioral videos before and after revision of the IBB operational definitions and score sheet**. **(A)** Three experienced raters and six novice raters participated in the first round of inter-rater reliability testing. Mean difference scores (MDS) from a “gold-standard” consensus score were calculated as described in the methods. Following score-sheet revisions, a second round of inter-rater reliability testing was performed by three experienced and seven novice raters. Note that the MDS values as well as their standard errors (SE) were reduced after the revisions, indicating an increase in inter-rater reliability. **(B)** Pearson correlations between the mean IBB score and the consensus score suggest a high degree of agreement with consensus in both novice and experienced raters, providing strong evidence that the IBB has high inter-rater reliability that improves with practice.

The results of the first IRR test (nine raters; three experienced, six novice) are shown in Figure 3 and present the MDS (i.e., the absolute value of the difference between the assigned score and the consensus or “gold standard” score) for ratings of performance shown in the 10 videos. Experienced raters scored within <1 point of the consensus score (0.8 ± 0.36) while novice raters scored within an average of 1.5 ± 0.5 points of the consensus score. This suggests that experienced raters independently assigning scores for the 10 videos are more accurate than novice raters, but novice raters could clearly get in the range of experienced raters with only a one-day training session. Correlational analysis of the separate expert inter-rater scores revealed significant reliability (all *r* values >0.9, *p* < 0.0001).

**Figure 4:**
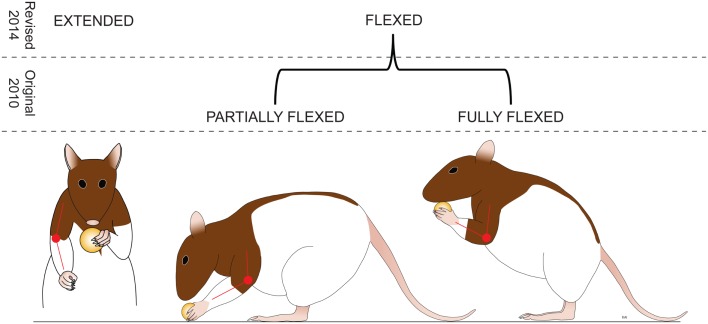
**Amendment: predominant elbow position**. The rat is assessed for the most common position (more than 50% of the time) assumed by the elbow during eating. Extended is when the elbow is held straight with an angle of more than 160°. *Flexed – The elbow is flexed with an angle of less than 160*°. (Revisions of the IBB scale from the JoVE 2010 version are highlighted in italics.)

**Figure 5:**
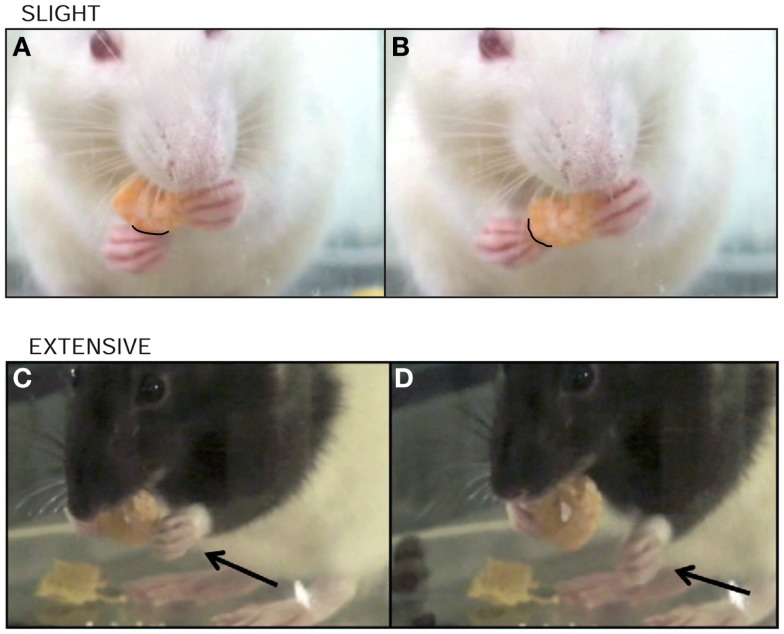
**Amendment: proximal forelimb movements**. The rat is assessed for movements made by the shoulder and/or elbow of the impaired forelimb that may or may not result in contact of the forelimb with the cereal. These proximal forelimb movements are defined as either: none – there are no shoulder and/or elbow movements of the impaired forelimb. Slight **(A,B)** is defined as *infrequent movements (<5% of the time)* through less than third the range of the shoulder and/or elbow joint; twitches and shrugs fall into this category. Extensive is defined as *frequent movements (>5% of the time)* by the impaired forelimb *OR movements* **(C,D)** *that are more than third the range of the shoulder and/or elbow joint*. In early recovery, these movements can be numerous and erratic. (Revisions of the IBB scale from the JoVE 2010 version are highlighted in italics.)

**Figure 6:**
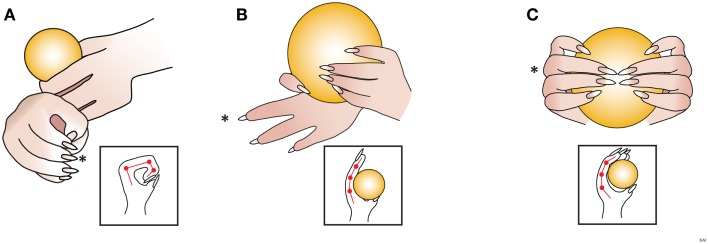
**Amendment: predominant forepaw position**. The rat is assessed for the most common position (more than 50% of the time) assumed by the digits. Scored as either **(A)** *clubbed flexed fixed – the digits are flexed and held in a fist with joint angles of about 90*°. **(B)** *Extended, non-adaptable – One or more of the digits are partially extended with joint angles between 180*° *and 160*°*; in addition, these digits DO NOT CONFORM to the shape of the cereal*. **(C)** *Partially extended, adaptable – digits are partially extended with joint angles between 160*° *and 90*°*; in addition, these digits CONFORM to the shape of the cereal*. Diagrams within the squares are observing the impaired forepaw, depicting digits 1 and 3 (*), from above. (Revisions of the IBB scale from the JoVE 2010 version are highlighted in italics.)

**Figure 7:**
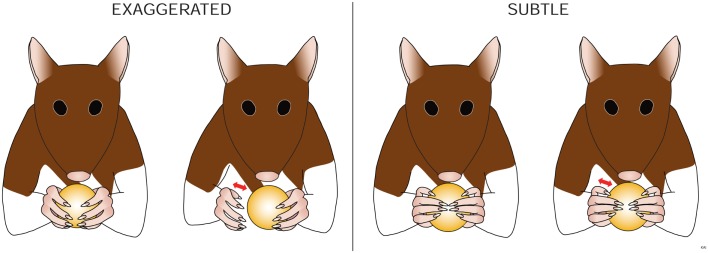
**Amendment: cereal adjustments (control)**. The rat is assessed for movements made by the impaired forelimb that are synchronized in time with successful manipulatory movements of the unimpaired forelimb, and that contribute to the proper manipulation of the cereal. These cereal adjustments can be defined as either: none – there are NO cereal adjustments made by the impaired forelimb. *Exaggerated – movements by the shoulder and/or elbow and/or wrist of the impaired forelimb that cause a loss of contact between the volar surface of the impaired forepaw and the cereal, which DO NOT adjust (control) the cereal position or DO NOT contribute to the proper manipulation of the cereal by the volar surface of the forepaws. Subtle – movements by the shoulder, and/or elbow, and/or wrist of the impaired forelimb that may or may not momentarily cause a loss of contact between the volar surface of the impaired forepaw and the cereal, which DO adjust (control) the cereal position or DO contribute to the proper manipulation of the cereal by the volar surface of the forepaws*. [If animals show both exaggerated and subtle proximal forelimb movements during eating, they are scored as having exaggerated movements, as these disappear with further recovery.] (Revisions of the IBB scale from the JoVE 2010 version are highlighted in italics.)

On review of the results by the group, a number of issues were identified that caused problems for the raters. These were:

1. The original scale rated the **Predominant Elbow Joint Position** as “extended, partially flexed, or fully flexed.” Discrimination between partially and fully flexed appeared to be problematic, and perhaps irrelevant in more recovered animals. Therefore, the predominant position subcategories were reduced to “extended” or “flexed” (Figure 4).
2. The definition for **Proximal Forelimb Movements** was initially defined only by the range of the movement; consideration of frequency of movements was identified as a feature that also reflected recovery and was deemed important to add to the operational definition. For example, many raters did not observe extensive movements in more well-recovered animals and thus scored the rat as 0 or 1, even though the rat was exhibiting a lot of recovery (Figure 5). Experienced raters appeared to ignore this aspect, so better clarification was warranted.
3. The explanation of the subcategory for Predominant Forepaw Position, **“Extended, Non-Adaptable,”** was unclear and needed more explanation. Participants also recommended that the designation of “Partially **Flexed** Adaptable” be changed to “Partially **Extended** Adaptable,” so the emphasis is on the recovery of extension (Figure 6).
4. The subcategories of **“Cereal Adjustments,”** “Exaggerated Movements,” and “Subtle Movements” needed further clarification as a distinction between these two levels was difficult. Momentary loss of contact, if the movement does contribute to proper cereal adjustment, was added to the explanation to increase discriminability (Figure 7).
5. Digit 5 was rarely visible. Elimination of the documentation of Digit 5 was recommended as it could not be consistently observed and scored.
6. A review of the participants’ data sheets revealed errors in score assignment. These errors were typically due to either ignoring a feature marked on the score sheet, or missing a feature required for a particular score. It was recommended that double-checking score assignments for accuracy be performed. The score determination guide also was revised to make scoring easier (Figure 2).

The revised IBB scale and definitions are shown in Table 1; the changes from that provided in Irvine et al. (1), are indicated by *italics* and underlining.

**Table 1:** ***Revised* IBB Forelimb Recovery Scale**.

**0:**	The predominant elbow position is EXTENDED, with NO or SLIGHT proximal forelimb movements and/or NO non-volar support by the forelimb ipsilateral to the injury site.
**1:**	The predominant elbow position is *FLEXED,* with SLIGHT proximal forelimb movements and SOME non-volar support by the forelimb ipsilateral to the injury site. The predominant forepaw position is CLUBBED, FIXED, and FLEXED.
**2:**	The predominant elbow position is FLEXED, with EXTENSIVE proximal forelimb movements and ALMOST ALWAYS non-volar support by the forelimb ipsilateral to the injury site. The predominant forepaw position is CLUBBED, FIXED, and FLEXED.
**3:**	The predominant elbow position is FLEXED, with EXTENSIVE proximal forelimb movements and NONE or SOME volar support by the forelimb ipsilateral to the injury. NONE or EXAGGERATED cereal adjustments are present. The predominant forepaw position is EXTENDED, NON-ADAPTABLE.
**4:**	The predominant elbow position is FLEXED, with EXTENSIVE proximal forelimb movements and SOME volar support by the forelimb ipsilateral to the injury site. EXAGGERATED cereal adjustments are present with NON-CONTACT movements of DIGIT 2 and possible wrist movements. The predominant forepaw position is EXTENDED, NON-ADAPTABLE.
**5:**	The predominant elbow position is FLEXED, with EXTENSIVE proximal forelimb movements and ALMOST ALWAYS volar support by the forelimb ipsilateral to the injury site. SUBTLE cereal adjustments are present with CONTACT MANIPULATORY movements of DIGIT 2 and possible wrist movements. The predominant forepaw position is EXTENDED, NON-ADAPTABLE.
**6:**	The predominant elbow position is FLEXED, with EXTENSIVE proximal forelimb movements and ALMOST ALWAYS volar support by the forelimb ipsilateral to the injury site. Wrist movements and SUBTLE cereal adjustments are present with CONTACT MANIPULATORY movements of DIGIT 2 and NON-CONTACT movements of DIGIT 3. The predominant forepaw position is EXTENDED, NON-ADAPTABLE with an ABNORMAL grasping method.
**7:**	The predominant elbow position is FLEXED, with EXTENSIVE proximal forelimb movements and ALMOST ALWAYS volar support by the forelimb ipsilateral to the injury site. Wrist movements and SUBTLE cereal adjustments are present with CONTACT MANIPULATORY movements of DIGIT 2 and 3 and NON-CONTACT movements of DIGIT 4. The predominant forepaw position is PARTIALLY *EXTENDED* but ADAPTABLE with a SOMETIMES NORMAL grasping method.
**8:**	The predominant elbow position is FLEXED, with EXTENSIVE proximal limb movements and ALMOST ALWAYS volar support by the forelimb ipsilateral to the injury site. Wrist movements and SUBTLE cereal adjustments are present with CONTACT MANIPULATORY movements of DIGITS 2, 3, and 4. The predominant forepaw position is PARTIALLY EXTENDED, ADAPTABLE with a SOMETIMES NORMAL grasping method.
**9:**	The predominant elbow position is FLEXED, with EXTENSIVE proximal limb movements and ALMOST ALWAYS volar support by the forelimb ipsilateral to the injury site. Wrist movements and SUBTLE cereal adjustments are present with CONTACT MANIPULATORY movements of DIGITS 2, 3, and 4. The predominant forepaw position is PARTIALLY EXTENDED, ADAPTABLE with an ALMOST ALWAYS NORMAL grasping method.
***REVISED* IBB DEFINITIONS**	
**Predominant elbow joint position:**	
The rat is assessed for the most common position (more than 50% of the time).	
	EXTENDED: The elbow is held straight with an angle *of >160°*.
	FLEXED: The elbow is flexed with an angle of *<160°*.
**Proximal forelimb movements:**	
The rat is assessed for movements made by the shoulder and/or elbow of the impaired forelimb that may or may not result in contact of the forelimb with the cereal.	
	NONE: There are no shoulder and/or elbow movements of the impaired forelimb.
	SLIGHT: *Infrequent* movements *(<5% of the time)* by the impaired forelimb through less than a third of the range of the shoulder and/or elbow. (Twitches and shrugs fall into this category.)
	EXTENSIVE: *Frequent* movements *(>5% of the time)* by the impaired forelimb *OR movements* that are greater than one-third of the range of the shoulder and/or elbow. In early recovery, these movements can be numerous and erratic.
Note: If animals show both slight and extensive proximal forelimb movements during eating they are scored as having extensive movements.	
**Contact non-volar support:**	
The rat is assessed for its ability to use the non-volar surface of the impaired forelimb to stabilize the cereal piece and in doing so, maintaining it in a position to aid eating. (Areas of the forelimb that may act as supports are the forearm above the wrist, the wrist or the back of digits.)	
	NONE: No non-volar support by the forelimb during eating (<5% of the time).
	SOME: Non-volar support of the object does occur during eating but not always.
	ALMOST ALWAYS: Non-volar support of the object occurs nearly always or always during eating (>95% of the time).
**Predominant forepaw position:**	
The rat is assessed for the most common position (more than 50% of the time) assumed by the digits, from flexed to extended, during eating.	
	CLUBBED, FLEXED, AND FIXED: Digits are flexed with joint angles greater than 90° and are held in a fist.
	EXTENDED, NON-ADAPTABLE: *One or more of* the digits are partially extended with joint angles between 180° and 160°; *in addition,* these digits *do not conform* to the shape of the cereal.
	PARTIALLY *EXTENDED,* ADAPTABLE: Digits are partially extended with joint angles between *160° and 90°; in addition,* these digits conform to the shape of the cereal.
**Contact volar support:**	
The rat is assessed for its ability to use the volar (palmar) surface of the impaired forepaw to stabilize the cereal and, in doing so, maintains a position to aid eating.	
	NONE: No volar support by the forelimb during eating (<5% of the time).
	SOME: Volar support of the object does occur during eating but not always.
	ALMOST ALWAYS: Volar support of the object occurs nearly always or always during eating (>95% of the time).
**Cereal adjustments (Control):**	
The rat is assessed for movements made by the shoulder and/or elbow and or/wrist of the impaired forelimb that are synchronized (in time) with successful manipulatory movements of the unimpaired forelimb, and that contribute to the proper adjustment (control) of the cereal position by the volar surface of both forepaws.	
	NONE: There are NO manipulatory movements made by the volar surface of the impaired forepaw.
	EXAGGERATED: Hypermetric movements of the shoulder and/or elbow and/or wrist of the impaired forelimb that:
Cause a loss of contact between the volar surface of the impaired forepaw and the cereal, ***and***	
DO NOT adjust (control) the cereal position or DO NOT contribute to the proper manipulation of the cereal by the volar surface of the forepaws.	
	SUBTLE: Tiny movements of the shoulder and/or elbow and/or wrist of the impaired forelimb that:
*May or may not momentarily cause a loss of contact between the volar surface of the impaired forepaw and the cereal, **and***	
DO adjust (control) the cereal position or DO contribute to the proper manipulation of the cereal by the volar surface of the forepaws.	
Note: If animals show both exaggerated and subtle proximal forelimb movements during eating, they are scored as having exaggerated movements, as these disappear with further recovery.	
**Wrist movements:**	
The rat is assessed for the presence of wrist movements of the impaired forepaw during eating, once volar support has been established. Movements of the wrist that occur in the absence of contact between the impaired forepaw and the cereal are *not* scored. These movements can occur in any direction, e.g., a dorsal (towards the back) to ventral (down towards the stomach) direction or medial (in towards the body midline) to lateral (away from the body midline) direction:	
YES	
NO	
**Presence of digit movements:**	
The rat is assessed for the presence of movements made by the individual digits during eating.	
	NON-CONTACT, YES or NO: Movements of the digits occur but these movements *do not* result in volar contact with the cereal.
	CONTACT MANIPULATORY, YES or NO: Movements of the digits occur that *do* result in volar contact of the digit with the object and, in doing so, contribute to manipulation of the cereal.
**Grasping method:**	
The rat is assessed for the most common (more than 50% of the time) grasping technique used during the eating phase. Several grasping methods exist but the most common are the “pincer,” the “hook,” and the “whole” grasp. The grasping techniques used by the rat are stereotypical depending on the size and shape of the cereal piece.	
	ABNORMAL: Consistent use of an alternative method of grasping to the method used prior to injury to support and control the cereal piece during the eating phase.
	SOMETIMES NORMAL: Inconsistent use of the grasping method used prior to injury to support and control the cereal piece during the eating phase.
	ALMOST ALWAYS NORMAL: Consistent use of the grasping method used prior to injury to support and control the cereal piece during the eating phase.

*The changes from that provided in Ref. (1), are indicated by *italics* and underlining*.

#### Inter-rater reliability test 2

After the changes were made, a second IRR test (three experienced, seven novice raters) was performed to determine if the changes increased clarity and thus accuracy. As shown in Figure 3, following the revisions, experienced raters had a mean difference from consensus score of 0.16 ± 0.15 points and novice raters had a MDS of 1.23 ± 0.05. Experienced observers continued to show more accurate ratings, but all raters increased accuracy. The revisions not only increased accuracy, but also reduced variability in score assignment and improved IRR as reflected by a reduction in the overall variability in score assignments. Improved accuracy is revealed by the reduction in deviation from the consensus score. In addition, Pearson correlations between each rater and the gold standard were consistently high (Figure 3B).

### Validity

#### Internal and face validity

**Figure 8:**
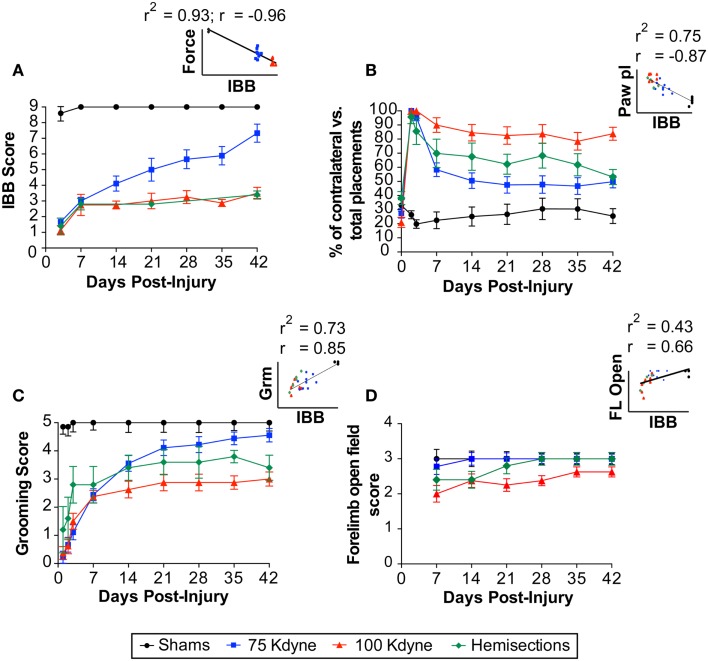
**Face, internal, and concurrent validity of the IBB score**. **(A)** Face and internal validity of the IBB score is provided by responsiveness to experimentally graded spinal cord injuries as well as the correlation (inset) with a biomechanical measurement of tissue displacement at the time of contusion injury. Concurrent validity is provided by comparisons with other established outcomes including **(B)** paw placement, **(C)** grooming score, and **(D)** forelimb open field. Insets reflect the scatterplot and regression line between the IBB and each of the established tests. The Pearson correlation (*r*) and the shared variance (*r*^2^) for each appear above the scatterplot; group identity for each point is color coded.

To assess internal and face validity of the IBB, we tested its sensitivity to a well-established experimental manipulation: graded SCI. We assessed sensitivity using a mixed repeated measures ANOVA (*F*-test) as well as effect size calculations (eta squared, η^2^). To assess the IBB’s sensitivity to recovery we performed repeated IBB testing over the post-injury interval. As shown in Figure 8A, the IBB was highly sensitive to the main effect of injury [sham, 75, 100 kdynes, or hemisection; *F*(3,24) = 120.89, *p* < 0.00001]. Effect size calculations indicated a very large effect of injury on IBB (η^2^ = 0.94), over six times higher than the classical definition of “large” effect size (0.14) (31). This indicates that the IBB was highly sensitive to the effect of SCI. The IBB also performed very well as a measure of recovery over time, *F*(3,72) = 27.52, *p* < 0.00001, η^2^ = 0.53. In addition, the IBB was highly sensitive to the injury × time interaction, *F*(9, 72) = 7.20, *p* < 0.00001, η^2^ = 0.47. The interaction term, in particular, indicates that the IBB is highly sensitive to the variable patterns of recovery produced by different SCI gradations. In addition, as shown in Figure 8A (inset), the IBB correlated very highly with the observed (“actual”) injury force biomechanical read-out from the IH device force transducer (*r* = −0.96; *r*^2^ = 0.93), providing strong evidence of face validity. Altogether these findings indicate that the IBB is an internally valid measure for assessment of recovery after SCI.

#### Concurrent validity: relationship to other functional tests

**Figure 9:**
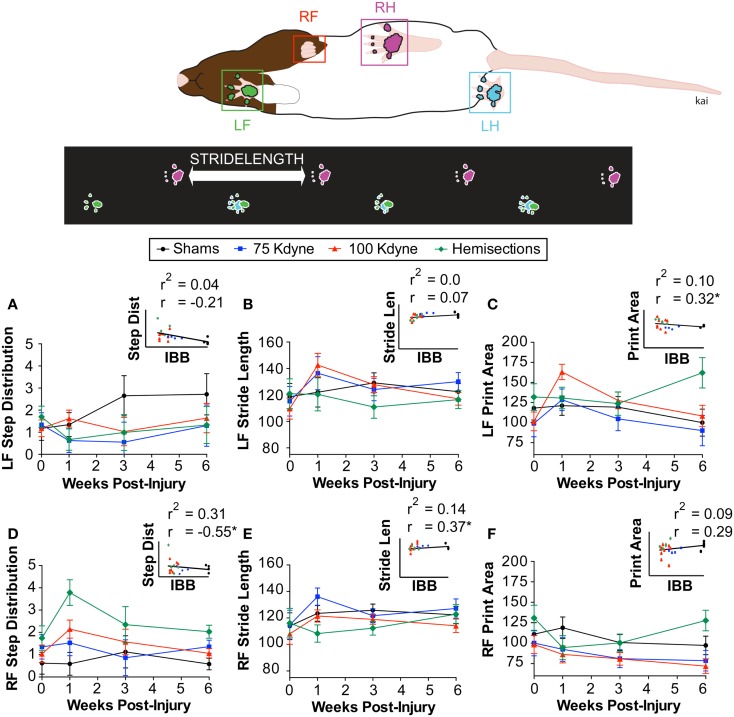
**Concurrent validity of the IBB with respect to automated gait analysis on the CatWalk**. **(A)** Left forelimb step distribution. **(B)** Left forelimb stride length. **(C)** Left forelimb print area. **(D)** Right forelimb step distribution. **(E)** Right forelimb stride length. **(F)** Right forelimb print area. Insets reflect the scatterplot and regression line between the IBB and each of the CatWalk outcomes. The Pearson correlation (*r*) and the shared variance (*r*^2^) appear above each scatterplot; group identity for each point is color coded. * Indicates significant correlation above *r*_crit_ = 0.317.

To assess concurrent validity, we compared the IBB to other established tests of outcome after SCI performed within the same subjects, i.e., the grooming task, paw placement in a cylinder, CatWalk, and forelimb use for over-ground locomotion in the open field (Figures 8B-D; Figure 9). The IBB demonstrated a similar overall pattern of recovery as other measures, however, with mild injuries (75 kdynes) it appeared to show less of an asymptotic performance ceiling in later recovery stages, suggesting that it may have greater sensitivity to continued recovery in high-functioning individuals. In addition, the IBB significantly correlated with paw preference asymmetry in the cylinder (Figure 8B, *r* = −0.87; *r*^2^ = 0.75), forelimb grooming test (Figure 8C, *r* = 0.85; *r*^2^ = 0.73), and forelimb open-field (Figure 8D, *r* = 0.66; *r*^2^ = 0.43). Comparisons to the CatWalk yielded less robust correlations (Figure 9), with significance reached (*r*_crit_ = 0.317) for the correlation with left (contralateral) forelimb print area (*r* = 0.32; *r*^2^ = 0.10), right (ipsilateral) forelimb step distribution (*r* = 0.55; *r*^2^ = 0.31), and right forelimb stride length (*r* = 0.37; *r*^2^ = 0.14). This reinforces prior work suggesting that only a subset of CatWalk measures are sensitive to the effects of unilateral cervical contusion injuries (2, 6). Altogether, the analytics reveal that the IBB has high concurrent validity.

#### Predictive validity: relationship to terminal histology

**Figure 10:**
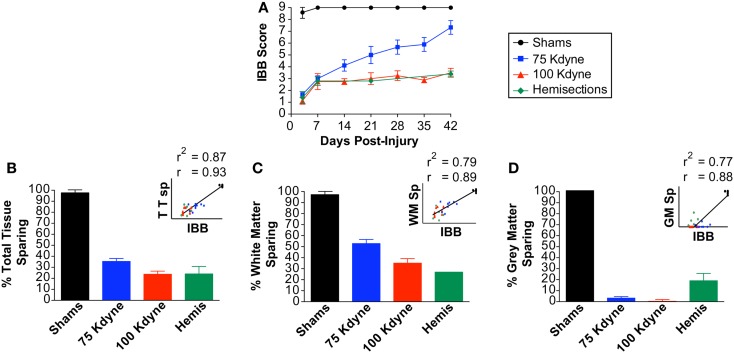
**Predictive validity of the IBB score with respect to histological outcome after spinal cord injury**. **(A)** IBB score. **(B)** Total tissue sparing at lesion epicenter. **(C)** White matter sparing at lesion epicenter. **(D)** Gray matter sparing at lesion epicenter. Insets reflect the scatterplot and regression line between the IBB (averaged over time) and each of the established tests. The Pearson correlation (*r*) and the shared variance (*r*^2^) appear above each scatterplot; group identity for each point is color coded.

To assess the predictive validity of the IBB test, we assessed its ability to predict postmortem histology (Figure 10). The IBB scores were averaged over the 42-day recovery interval and the binned IBB scores were correlated with postmortem histopathological assessment of total tissue sparing, white matter sparing, and gray matter sparing and motor neuron counts. The results revealed significant correlations for each of these measures (*r* = 0.93, *r*^2^ = 0.87; *r* = 0.89, *r*^2^ = 0.79; *r* = 0.88, *r*^2^ = 0.77; *r* = 0.68, *r*^2^ = 0.46, respectively; Figure 10, insets). Together, these results suggest that the IBB is highly predictive of histological changes after SCI, providing strong support for its use as a behavioral biomarker for SCI outcome assessment.

Correlations of individual variables with the IBB score were done using all animals including the shams. The reason for this was that we wanted the entire range of behavior and anatomy to be represented (i.e., from most injured with no function to no injury and normal function). An alternative approach is to ask if the scale is sensitive within the range of injury and partial function, i.e., without the shams. Table 2 presents the correlations figured both ways. Pearson correlations (*r*) and shared variance (*r*^2^) deflated without shams, indicating a smaller but often still significant dynamic range within different injury conditions. This suggests that the IBB has sensitivity across a wide dynamic range of injury conditions. Note that *r*_crit_ = 0.31 for *p* < 0.05.

**Table 2:** **Correlations of individual variables with IBB score**.

Variable	*r* (all subjects)	*r*^2^ (all subjects)	*r* (no shams)	*r*^2^ (no shams)
Actual force	−0.96	0.93	−0.75	0.56
Tissue displacement	−0.83	0.70	−0.09	0.01
Abnormal paw PL	−0.87	0.75	−0.69	0.48
Grooming	0.85	0.73	0.47	0.22
Forelimb open field	0.66	0.43	0.67	0.45
LF step distribution	−0.21	0.04	−0.31	0.10
LF stride length	0.07	0.00	0.34	0.12
LF print area	0.32	0.10	0.42	0.17
RF step distribution	−0.55	0.31	−0.27	0.08
RF stride length	0.37	0.14	0.67	0.45
RF print area	0.29	0.09	0.03	0.00
Total sparing	0.93	0.87	0.55	0.30
WM sparing	0.89	0.79	0.61	0.37
GM sparing	0.88	0.77	0.06	0.00
Motorneuron sparing	0.68	0.46	0.27	0.07

*Note that separate correlations were calculated for all injury conditions (all subjects) and excluding shams (no shams). Note that Pearson correlations (*r*) and shared variance (*r*^2^) deflated without shams, indicating a smaller but often still significant dynamic range within different injury conditions. This suggests that the IBB has sensitivity across a wide dynamic range of injury conditions. Note: *r*_crit_ = 0.31 for *p* < 0.05*.

#### External validity: responsiveness to other types of neurological injuries

**Figure 11:**
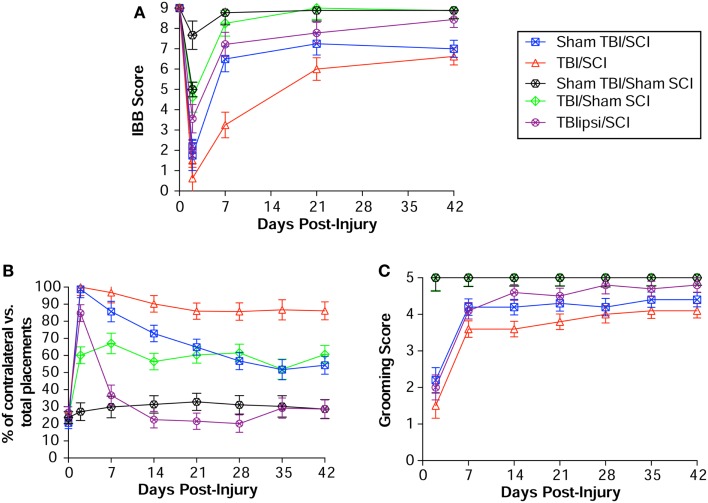
**External validity of the IBB Score**. **(A)** The IBB was performed in an independent cohort of subjects as part of a model-development project for spinal cord injury (SCI) with concomitant traumatic brain injury (TBI). Note that the IBB was sensitive to the impact of SCI as well as the additive effect of SCI + TBI. **(B)** Paw placement and **(C)** grooming in the same subject cohort for comparative purposes. Reprinted with permission from Ref. (24).

To assess whether the IBB has external validity, we tested a new population of subjects and also assessed its sensitivity to alternative forms of neurological injury in the context of a model-development effort for central nervous system (CNS) polytrauma (SCI + TBI; (24)). IBB was assessed in subjects receiving either a unilateral cervical SCI alone (75 kdynes), TBI alone, or SCI + TBI combined injuries (with the TBI either ipsilateral or contralateral to the SCI). If the IBB has high external validity then it should show graded sensitivity in this new population of subjects. The results are shown in Figure 11, and demonstrate that IBB was highly sensitive to the impact of injury condition, *F*(4,37) = 15.74, *p* < 0.00001. The sensitivity of the IBB to CNS injury was reinforced with a very large effect size η^2^ = 0.63, over four times higher than the classical cut off for “large” effect size [η^2^ = 0.14; (31)]. Together, the results indicate that the IBB has high external validity for the combinatorial effect of SCI + TBI. Note, that the IBB was selectively sensitive to the impact of TBI contralateral to the SCI, but little impacted by TBI alone. This suggests that the IBB, like the grooming test, is somewhat selective for the effects of SCI, and perhaps, selectively sensitive to anatomical substrates through which contralateral cortical contusion impacts SCI recovery [see Ref. (24), and “Discussion” section for further review).

#### Construct validity: multidimensional syndromic assessment

Spinal cord injury is an intrinsically multifaceted syndrome that can be conceptualized within a multivariate, big-data analytic framework (2, 32–37). In this context, we can assess construct validity of SCI outcome batteries by borrowing well-established methods from the educational and neuropsychiatric testing fields. Namely, we can apply multivariate exploratory factor analysis on the full set of multi-trait multi-method outcomes to derive the underlying latent structure of the SCI syndromic space (2, 29, 38, 39). This approach is a realization of classical arguments about strong inference and the need to leverage full-information to deal with complexity in biology and neuroscience (40).

**Figure 12:**
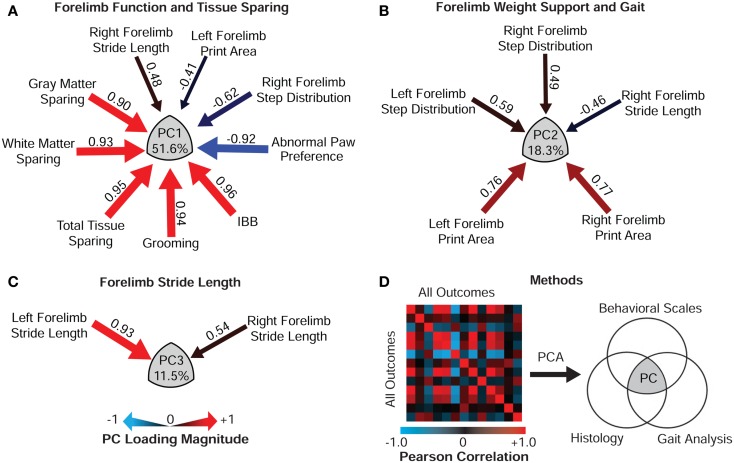
**Construct validity of the IBB Score**. Principal component analysis (PCA) extracted three orthogonal multivariable principal component (PC) clusters that together accounted for 81.4% of the variance in outcome after SCI. **(A)** PC1, the largest cluster of variance (51.6%) reflects the relationship between forelimb function and histological outcome. Note the IBB score is the highest loading variable on PC1, providing evidence of construct validity. **(B)** PC2 (18.3% variance) reflected the relationship of forelimb weight support and gait. **(C)** PC3 (11.5% variance) reflects forelimb stride length. **(D)** PCA extracts the PCs through eigenvalue decomposition of the bivariate correlation matrix of all outcomes, here represented as a heat map of Pearson values. PCs are reflected as the Venn intersection (gray) across outcome domains and the PC loading values (correlation between each variable and the PC cluster) are represented as arrows where gage represents loading magnitude and heat reflects direction (red positive relationship, blue inverse relationship).

To assess the relationship of the IBB to multidimensional SCI, we performed exploratory factor analysis using the extraction method of PCA. PCA integrates the full bivariate cross-correlation matrix of all biological and functional outcomes through multivariate pattern detection coupled with dimension-reduction ((2, 29); Figure 12). In essence, PCA reduces the total number of observed variables down to a small number of principal components (PCs; or “latent variables”) that concisely summarize the overall set of observations within the dataset. We performed PCA on the full set of outcome variables presented (in univariate form) in Figures 8–11. PCA revealed three latent multivariables (PC 1–3) that together accounted for 81.4% of the variance in outcome (Figures 12A–C). To understand how individual outcome metrics relate to the PC syndromic patterns, we plotted the correlation (so called “loadings”) of each outcome metric on the PC patterns. Significant loadings above 0.45 are represented as arrows where arrow size indicates magnitude and heat represents valence (positive vs. inverse relationships). Note that IBB loads very highly on PC1, indicating that it is a highly de-noised measure of the latent construct represented by PC1. As in prior work (2), the PC1 loading pattern suggests that it represents the relationship between tissue sparing and recovery of function – the multidimensional target for neuroprotective therapies. The fact that the IBB is the highest loading variable on PC1, suggests that it is a powerful surrogate biomarker for the set of variables represented by PC1. In addition, note that IBB does not load on PC2 or PC3, which are both devoid of histological loadings. This suggests that the IBB is a highly selective detector of the histopathology–behavior relationship. Combined with the univariate validity testing, the multivariate results provide strong validation of the IBB as a measure of recovery of function following cervical SCI.

## Discussion

### Development of the IBB

A major goal of preclinical modeling for SCI is to identify methods that can be used to evaluate treatments for translation to clinical trials. Our prior work on cervical SCI (6) used a variety of tasks to measure forelimb function including the grooming task, paw placement in a cylinder, CatWalk, and forelimb open-field locomotion. It is noteworthy that these tasks largely assessed proximal forelimb movements with some limited information about hand use. None of these tests focused on digit function, which we consider to be important to assess for the translational relevance of our preclinical outcome testing. A number of tasks that assess distal forelimb movements in rodents have been described especially by Whishaw and colleagues, and many have focused on the “reach-to-grasp task” [reviewed in Ref. (41)]. This task however, requires extensive training and food deprivation. We also considered an alternative task, pasta eating, that required hand use to accommodate a variety of food shapes (17, 18) and was sensitive to forebrain injuries. However, during the process of trying to acclimate rats to a variety of food items, we noticed that acutely injured subjects demonstrated movements of the affected limb during eating that did not contribute to food manipulation. The hand was fixed in a fisted position preventing the digits from grasping the food, and the forelimb was only used to support the food item. In contrast, the contralateral limb showed fine digital movements. Allred and colleagues (17) had made similar observations in their description of the “Vermicelli handling task,” in which rats are filmed eating pieces of thin pasta and manipulation of the pasta was compared to pre-injury handling methods. However, the juxtaposition of the digits during pasta eating made it difficult to discern movement of individual digits, and only movements with physical contact with the pasta were described and assessed. We considered that this strategy would ignore the rats’ attempts to use the forepaw ipsilateral to the SCI, and its continued improvement over time.

We therefore explored developing a formal observational scale to rate recovery of both proximal and distal forelimb movements in the affected limb during food manipulation, including fine digital control. Using a high-definition camera, we filmed subjects eating consistently sized cereal pieces in a Plexiglas cylinder surrounded by mirrors to enable 360° viewing of the movements. Both uninjured subjects and subjects with a range of unilateral cervical injuries produced by the IH device were examined over 6 weeks. Initial observations were unconstrained notes based loosely on the structured note-taking scheme of the BBB locomotor rating scale (19). Like the BBB, attention was first given to gross position of the joints in the affected limb and then to more refined features of movement. We also noted differences in the grasping techniques across different cereal shapes, largely inspired by work of Whishaw and colleagues. The result of this analysis, termed the “IBB,” was described in Irvine et al. (1).

In the current paper, we have assessed this method for both reliability and validity. These are distinct but related issues in the field of testing theory. IRR deals with the issue of consistent scoring of observations whereas validity deals with the issue of whether a measurement assesses what it purports to assess. These issues will be discussed separately below.

### Inter-rater reliability

Inter-rater reliability deals with whether an assessment tool is consistent from rater to rater. To assess IRR, we used an approach similar to that used during the development of the BBB Locomotor Rating Scale (21). This approach relied on assessing deviations from a gold-standard consensus score that is derived by expert raters working together as a team. The current study used a consistent set of videos to assess IRR. This provided some advantages over the live-rating strategies used to assess the BBB scale. First, it ensured that there was only one view of the behavior, providing a more direct assessment of inter-rater variability. Second, we could randomize the presentation of the exact same behavior allowing us to control for sequence effects in raters. We found that there was a high concurrence of score assignment for both experienced and novice raters, and that concurrence was improved after some minor adjustments to the scale definitions and procedures. We also found that experience improves consistency and accuracy of score assignment [as was observed with the BBB; Ref. (21)]. Novice raters could be trained to identify the behavioral features for rating within a single day, and were able to identify definitional issues that, when changed, improved accuracy for both novice and experienced raters. The full set of IRR assessment videos and materials are available to qualified neurobiological researchers upon request. Given that the videos are identical, researchers should be able to match their results to those presented in the current paper.

### Internal/face validity

The internal or face validity of this measure is reflected in its ability to detect differences in the degree of injury to the nervous system. Performance in cohorts of animals with 75 and 100 kdyne unilateral contusion SCI, lateral hemisection, and combined SCI with TBI showed that the IBB was sensitive to varying damage to the spinal cord and cortex, both individually and in combination. Graded SCI produced differential recovery (Figure 8A). Interestingly, TBI alone produced a mild initial deficit which quickly recovered (by 1 week post-TBI; Figure 11, green line). Whishaw et al. (42) showed that cortical lesions did not affect the ability of rats to pick up food with their mouth and transfer it to their hands for manipulation, but did observe that cortical injuries produced difficulty with pronation and supination. This type of deficit could be reflected in the early mild suppression of the IBB score after the cortical injury alone. Interestingly, the addition of a cortical injury contralateral to an SCI, produced a significant depression of IBB scores over the SCI alone, suggesting that the contralateral cortex was involved in the recovery from the SCI. A TBI placed ipsilaterally to the SCI, did not show the same effect as the contralaterally placed TBI, and in fact slightly, but not significantly, improved outcome on this measure. The dual lesions’ effect on the circuitry supporting paw use is complex and a multivariate approach to determining the output shows that this is indeed the case (35) but is beyond the scope of the present discussion.

### Concurrent validity

Concurrent validity asks how performance on this test relates to performance on other tests used to assess recovery after unilateral SCI [e.g., Ref. (4, 6, 9)]. The current study found that IBB scores correlate very highly with paw placement and grooming scores, and less highly, but still significantly, with forelimb use for locomotion in the open field and on the Catwalk (although only on some of the Catwalk measures). These tests evaluate hand use during vertical exploration, during grooming of the face and head, and for locomotion respectively. Other tests which evaluate hand use during grasp and retrieval [e.g., Ref. (42–44)] were not tested. The IBB test focuses on a different aspect of forelimb use than the reach and grasp tasks. The IBB represents an assessment of hand use during food manipulation for consumption as opposed to reaching and grasping tasks, which involve forelimb use for retrieval of items distal to the animal (41, 45). During reaching tasks, animals are required to extend their arm through a slot to reach a food object. The hand is then brought over the food pellet using a stereotyped arpeggio movement and the pellet is grasped, followed by bringing the food to the mouth. For the IBB, animals first locate the food on the floor of the cage using at least olfaction and somatosensory input via the vibrissae, they pick the food up with their mouth and then bring the forelimbs to the mouth to support and manipulate the food, especially if the item is large. The food is then rotated and positioned for biting with both hands. The reach and grasp tasks do not focus on this proximal manipulation during consumption. In this sense the IBB is complementary to reach and grasp tasks.

Whishaw has pointed out that “reach and grasp” is a highly evolutionarily conserved function that is similar across the mammalian class, and thus is likely to be a useful tool for translational modeling (41). While the ability to use fine digital movements increases and individuates as one “ascends” the class from rodents to primates, the basic organization of the neural systems underling these behaviors are likely to be similar. Therefore, attempts to develop outcome measures with similar features across species that can be combined to develop batteries of tests evaluating different substrates for recovery, would seem to increase the probability of translation from rodent injury models to the human clinical situation. In this sense, the IBB represents an important addition to a complete battery of tests that can be used to assess recovery of function after cervical SCI. By combining data from multiple tests, we will have a better, more holistic view of recovery after neurological injury.

### Predictive and external validity

To test the predictive validity of the IBB, we examined the relationship with the underlying tissue damage in the spinal cord. We found that the IBB scores were highly and significantly correlated with the amount of tissue sparing at the SCI lesion site. How the IBB predicts SCI severity in comparison to other tests is discussed in the multivariate section below. The IBB was minimally sensitive to the impact of TBI alone, but as mentioned above, showed a similar sensitivity to combined SCI + TBI as the paw placement test (24). In a recent report from Speck et al. (46), the IBB was also shown to be sensitive to recovery from peripheral nerve injuries in mice.

### Construct validity: Multivariate assessment of function

Findings from multifaceted outcome batteries applied to the same subject ultimately need to be integrated in some manner to derive a complete picture of forelimb recovery. Multivariate statistical pattern detectors such as PCA and the related approach of exploratory factor analysis provide quantitative means to perform this integration across outcomes (29, 39). This approach has classically been applied in the human assessment literature as a tool to gauge construct validity: the degree to which an individual test measures or “taps into” an underlying trait of interest [e.g., intelligence, executive function, memory etc.; Ref. (39)]. Indeed, this application of multivariate statistics is the underlying basis for most modern, standardized human achievement and neuropsychological tests. However, PCA has rarely been applied in preclinical research studies to assess the validity of scales used in animal models of neurobiological disorders. In the present paper we applied PCA to, (1) integrate outcome across multiple assessment tools, and (2) to assess the construct validity of the IBB. Based on prior work, we knew that PCA has the capacity to detect specific neurobiological substrates for forelimb recovery after SCI, specifically tapping into the relationship between tissue sparing and multifaceted forelimb function on the first principal component (PC1) (2, 32, 33, 37). The question in the current paper was, “does the IBB predict (or “load onto”) the established forelimb neurobehavioral recovery construct outcome set?” The results indicated that not only did the IBB predict the forelimb neurobehavioral recovery construct (PC1), but it actually had the highest loading of all of the outcome variables assessed, providing strong evidence of construct validity for the IBB.

It is noteworthy that the IBB did not correlate as well with CatWalk measures of gait during locomotion. This suggests that the CatWalk assesses different neurobiological substrates than the IBB. This is consistent with prior work showing that the CatWalk outcome metrics do not have high construct validity with respect to multivariate tissue sparing in contusive SCI (PC1) but do tap into orthogonal variance (PC2, PC3) related to hemisection injuries (2). This indicates that the CatWalk may reflect tissue changes not captured by crude measures of histological sparing after unilateral cervical SCI. This could account for the observation that hemisection injuries impact CatWalk, a model in which white matter and gray matter sparing at the lesion epicenter are relatively consistent. This dissociation between CatWalk and tissue sparing is reminiscent of the pattern observed in prior analyses that have included the horizontal ladder test after cervical SCI (6, 47). The horizontal ladder, the CatWalk and forelimb locomotor function clustered together as a coherent functional assessment construct (PC2); however, this outcome cluster did not correlate with histological sparing (47). We have argued that this indicates that CatWalk and horizontal ladder reflect fine-details of locomotor recovery that are organized by more subtle neurobiological changes (perhaps due to sprouting and plasticity), not reflected by gross gray and white matter sparing metrics *per se* (2, 37).

### Forelimb object manipulation as a translational tool

Our group has begun developing a primate analog to the IBB to facilitate cross-species translation of SCI research findings (34, 48, 49). Early work suggests that the IBB can be scaled up into an analogous object manipulation task in a non-human primate (NHP) model of cervical SCI in the rhesus macaque (48, 49). The primate version of the task shows strong sensitivity for loss and recovery of function after cervical lateral hemisection injuries. In addition, early cross-species testing of construct validity suggests that the rodent IBB and primate object manipulation task co-load along with tissue sparing on PC1, enabling consistent assessment of translational features of forelimb recovery (34, 48, 49).

Of course, the utility of object manipulation as a translational outcome measure may depend on the neurobiological substrates under study. It is often assumed that much of the loss and recovery of fine digital movement, and reach and grasp, in humans after CNS damage or degeneration is due to loss of cortico-spinal tract (CST) function. The classic work of Lawrence and Kuypers (50–52) indeed points to the pyramidal tract as a critical mediator of forelimb and especially fine digital control in primates. However, attempts to assign specific roles to the multitude of descending tracts and intra-spinal circuits in experimental models of SCI have proven to be difficult, and recent work suggests that there may be considerable redundancy in the organization of forelimb motor function. For example, Fouad and colleagues tested performance on a single pellet reaching task after various lesions of the dorsal and lateral funiculi, and found little correlation between lesion size and performance in the rat (53). In a related study, Morris et al. (54) found that lesions restricted to the dorsolateral funiculus where the rubrospinal tract is located, only affected the “arpeggio” movement, and not other aspects of reach and grasp.

It seems clear that more flexibility and individuation of movement might be supported by the development of the cortical system mediated through the CST as the primate CST developed, and that the ability of primates to produce highly accurate ballistic movements in space and to produce individual finger movements is extraordinary. However, recent work from several laboratories using NHPs suggests that recovery of fine digital control can be accomplished via reorganization of descending reticular systems impinging upon interneurons in the cervical cord. This raises the issue of how much of the forelimb control is mediated by cortical brainstem circuits versus those organized intrinsically within the cervical cord. In the case of the IBB scale, the results of our CCI studies suggest that the circuits in the sensorimotor cortex are involved in recovery of forelimb and fine digital movements, but that certainly much of this circuitry is organized at the spinal level, at least in the rodent.

Comparative studies of the neurobiology of forelimb recovery after rodent and primate SCI are a major focus of ongoing studies (55, 56). Object manipulation tasks such as the IBB will play an important role in making these cross-species comparisons to unravel the neurobiological substrates of forelimb recovery in the context of translational therapeutic testing.

## Conclusion

The IBB is a recently developed forelimb scale for the assessment of fine control of the digits after damage to the nervous system (1). The present paper suggests that the IBB has strong IRR and validity (face, concurrent, and construct). Thus, the IBB may be useful in conjunction with, and in comparison to, other measures of forelimb and fine digital control in other mammalian species including primates. And, it may be a valuable adjunct to the armamentarium of translational tools for assessing recovery after nervous system damage and degeneration.

## Author Contributions

Karen-Amanda Irvine, Stephanie B. Beattie, Jacqueline C. Bresnahan, Michael S. Beattie provided the testing concept, Karen-Amanda Irvine, Stephanie B. Beattie, Jacqueline C. Bresnahan, Adam R. Ferguson, Michael S. Beattie designed the studies; all authors participated in the data collection, IRR testing (except Stephanie B. Beattie), and the interpretation of the results; Adam R. Ferguson supervised the data analysis; Karen-Amanda Irvine implemented the figures with input from Adam R. Ferguson, Jacqueline C. Bresnahan, and Michael S. Beattie; Karen-Amanda Irvine, Adam R. Ferguson, Jacqueline C. Bresnahan, and Michael S. Beattie generated the manuscript draft, and all authors critically reviewed and revised the manuscript.

## Conflict of Interest Statement

The authors declare that the research was conducted in the absence of any commercial or financial relationships that could be construed as a potential conflict of interest.
